# Surfactant therapies for pediatric and neonatal ARDS: ESPNIC expert consensus opinion for future research steps

**DOI:** 10.1186/s13054-021-03489-6

**Published:** 2021-02-22

**Authors:** Daniele De Luca, Paola Cogo, Martin C. Kneyber, Paolo Biban, Malcolm Grace Semple, Jesus Perez-Gil, Giorgio Conti, Pierre Tissieres, Peter C. Rimensberger

**Affiliations:** 1 Division of Pediatrics and Neonatal Critical Care, “A.Béclère” Medical Centre, Paris Saclay University Hospitals, APHP, 157 Rue de la Porte de Trivaux, 92140 Clamart (Paris-IDF), France; 2 Physiopathology and Therapeutic Innovation Unit-INSERM U999, Paris Saclay University, Paris, France; 3 Department of Pediatrics, University of Udine, Udine, Italy; 4 Division of Pediatric Critical Care Medicine, Department of Pediatrics, Beatrix Children’s Hospital Groningen, University Medical Centre Groningen, University of Groningen, Groningen, The Netherlands; 5 Critical Care, Anesthesiology, Peri-Operative and Emergency Medicine (CAPE), University of Groningen, Groningen, The Netherlands; 6 Department of Neonatal and Pediatric Critical Care, Azienda Ospedaliera Universitaria Integrata Verona, Verona, Italy; 7 Health Protection Research Unit in Emerging and Zoonotic Infections, Department of Clinical Infection, Microbiology and Immunology, University of Liverpool, Liverpool, UK; 8 Department of Biochemistry and Molecular Biology and Research Institute “Hospital 12 de Octubre”, Complutense University, Madrid, Spain; 9 Department of Anesthesiology and Intensive Care, Catholic University of the Sacred Heart, Rome, Italy; 10 Division of Pediatric Critical Care and Neonatal Medicine, “Kremlin-Bicetre” Medical Center, Paris Saclay University Hospitals, APHP, Paris, France; 11 Integrative Cellular Biology Institute-UMR 9198, Host-Pathogen Interactions Team, Paris Saclay University, Paris, France; 12 Division of Neonatology and Pediatric Critical Care, Department of Pediatrics, University Hospital of Geneva, University of Geneva, Geneva, Switzerland

**Keywords:** Neonate, Infant, Children, PARDS, NARDS, Surfactant

## Abstract

Pediatric (PARDS) and neonatal (NARDS) acute respiratory distress syndrome have different age-specific characteristics and definitions. Trials on surfactant for ARDS in children and neonates have been performed well before the PARDS and NARDS definitions and yielded conflicting results. This is mainly due to heterogeneity in study design reflecting historic lack of pathobiology knowledge. We reviewed the available clinical and preclinical data to create an expert consensus aiming to inform future research steps and advance the knowledge in this area. Eight trials investigated the use of surfactant for ARDS in children and ten in neonates, respectively. There were improvements in oxygenation (7/8 trials in children, 7/10 in neonates) and mortality (3/8 trials in children, 1/10 in neonates) improved. Trials were heterogeneous for patients’ characteristics, surfactant type and administration strategy. Key pathobiological concepts were missed in study design. Consensus with strong agreement was reached on four statements:

1. There are sufficient preclinical and clinical data to support targeted research on surfactant therapies for PARDS and NARDS. Studies should be performed according to the currently available definitions and considering recent pathobiology knowledge.
2. PARDS and NARDS should be considered as syndromes and should be pre-clinically studied according to key characteristics, such as direct or indirect (primary or secondary) nature, clinical severity, infectious or non-infectious origin or patients’ age.
3. Explanatory should be preferred over pragmatic design for future trials on PARDS and NARDS.
4. Different clinical outcomes need to be chosen for PARDS and NARDS, according to the trial phase and design, trigger type, severity class and/or surfactant treatment policy*.*

We advocate for further well-designed preclinical and clinical studies to investigate the use of surfactant for PARDS and NARDS following these principles.
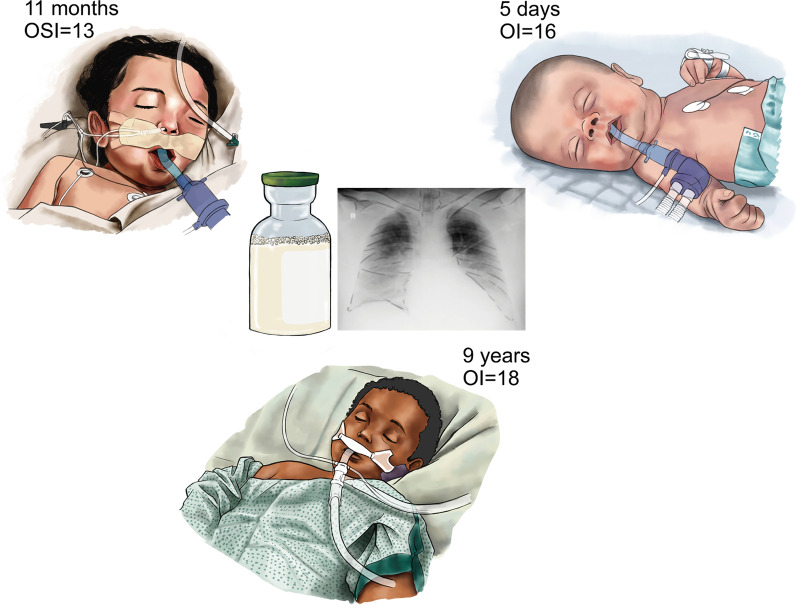

## Background

Acute respiratory distress syndrome (ARDS) occurs at any age including pediatric (PARDS) and neonatal (NARDS) patients, and age-specific definitions of the syndrome are now available, namely the pediatric acute lung injury consensus conference (PALICC) and the Montreux definition for PARDS and NARDS, respectively [1, 2]. PALICC and Montreux definitions should be used, respectively, for children beyond the first month of age and for neonates from birth until 4 weeks, or 44 weeks post-menstrual age if born before 40-weeks’ gestation (Additional file 1) [3]. ARDS is a life-threatening respiratory failure characterized by lung tissue inflammation [4] and alveolar and/or endothelial damage [5] coupled with a complex surfactant injury [6]. In children and neonates, these features may translate in different epidemiology, triggers and clinical approach [1, 7, 8]. For example, direct or pulmonary (primary) and indirect or extrapulmonary (secondary) ARDS may have peculiar triggers in children and neonates, such as meconium aspiration, bronchiolitis or necrotizing enterocolitis. Lung tissue in children is less prone to inflammation and fibrosis, while the relative volume is smaller as the alveolarization is still ongoing [9]. Endogenous surfactant is present in relatively higher concentrations, and similarly, higher doses of exogenous surfactant are more easily administered [9].

Despite these peculiarities, ARDS pathobiology pattern (inflammation, cellular damage and surfactant dysfunction) is the same in patients of any age. Inflammation and cellular damage also contribute to surfactant qualitative and quantitative injury, affecting both surfactant proteins and phospholipids [10]. From a molecular point of view, phospholipases, proteases, inflammatory mediators and oxygen reactive species play a role in this damage [3, 7]. Once the injury has been triggered, a vicious cycle drives ARDS: (1) phospholipase-driven surfactant phospholipid hydrolysis; (2) production of free fatty acids; and (3) derived inflammatory mediators; (4) further surfactant damage due to inflammatory mediators [12]. This ‘surfactant catabolism-inflammation-surfactant catabolism’ mechanism can perpetuate the injury and worsen the loss of alveolar-capillary barrier function. This, in its turn, facilitates the cellular influx toward the lung and the accumulation of proteins within the interstitial and alveolar space. Neutrophils and alveolar macrophages may increase the local inflammation, worsening the vicious cycle. In fact, in vivo studies on adult ARDS patients showed that disaturated phosphatidylcholine turnover is faster compared to controls, due to increased surfactant catabolism [13]. These mechanisms represent the ‘exudative phase’ of ARDS. They cause an increase in alveolar surface tension and a reduction in lung compliance. This pathobiology is known to be different between direct and indirect ARDS as the two types of the syndrome have distinct cellular and biochemical injury profile [14–16]. Data in pediatric patients are more limited but consistent and also indicated differences between ARDS of infectious or non-infectious origin [17]. Later, in the severest cases, the picture may eventually evolve into the ‘fibro-proliferative phase’ characterized by the formation of hyaline membranes, influx of proliferating mesenchymal cells, epithelial cell necrosis/apoptosis, thrombogenesis in pulmonary vessels and lung fibrosis. This phase may occur within 3–4 days of injury in some parts of the lung and can overlap with the exudative phase in other parts of the lung. Given this pathobiology, it is not surprising that surfactant has been considered a potential therapeutic agent for the early phase of ARDS, to reverse the surfactant dysfunction, improve compliance and (re-)open the alveoli, allowing better gas exchange and/or less aggressive respiratory support.

So far, attempts to cure ARDS with surfactant have not led to impressive results. This was likely due to many reasons such as: 1) lack of efficient alveolar surfactant delivery; 2) inactivation of surfactant by phospholipase A2 and other surfactant-injuring agents; 3) insufficient dose; 4) wrongful trial design. This latter may be due to several issues, but undoubtedly a main one is represented by lack of homogeneity as trials included very different patients, types of ARDS, surfactant preparations, comorbidities and co-interventions [18]. There is effort to find ‘enrichment tools’ (such as biomarkers or imaging techniques) to better describe ARDS populations in adult critical care [19], and this is definitively needed for patients of any age.

Several studies on the use of surfactant for ARDS in children and neonates have been published. Nonetheless, despite the pediatric/neonatal experience being wider than that of adult medicine, evidence in favor of surfactant is still unavailable [20]. We aim to analyze data to offer an expert consensus opinion suggesting how to advance the knowledge in pediatric (PARDS) and neonatal (NARDS) ARDS field.

## Methods

We planned to perform a narrative review about surfactant for ARDS in children and neonates, and based on this, to create a consensus opinion about future research steps in this field. The European Society for Pediatric and Neonatal Intensive Care (ESPNIC) created an expert committee, including two intensivists skilled in relevant methodology, as in previous ESPNIC guidelines [21]. The project was divided into two phases: (1) review of the literature and (2) production of expert consensus opinion. Since well-known trials’ heterogeneities prevent data aggregation [20], we did not perform any quantitative meta-analysis or bias assessment. This is consistent with our aim which was not to give any advice on clinical efficacy. We focused: on (1) any translational or animal study on surfactant biology during ARDS and (2) randomized controlled trials comparing surfactant therapy versus standard care for ARDS in children and neonates, irrespective of definition used to diagnose the syndrome. We decided so because PARDS and NARDS definition are quite recent; thus, many clinical studies had to be based on different entry criteria. Studies about the first-generation, protein-free surfactants have been excluded as they are no longer marketed and were not used in ARDS trials. These surfactants are clinically inferior to animal surfactant preparations, which contain higher amount of proteins, in preterm neonates with respiratory distress syndrome (RDS, i.e., hyaline membrane disease due to primary surfactant deficiency) [22]. Moreover, ARDS patients also show decreased levels of surfactant proteins; thus, protein-free surfactants are likely to be less efficacious to treat ARDS, as well [23]. Quaker-based technique [24] was used for panel discussion, and recommendations were voted as in previous ESPNIC guidelines [21]. Panel composition, literature review and consensus methodology are detailed in Additional file 2.

## Results

### Literature review

Additional file 3 reports the basic data of clinical trials included in the review. Trials were very heterogeneous for patients’ age, type of ARDS, entry criteria and case mix, surfactant type, dose and administration technique, injected volume and ventilatory policy. These are main points that may have prevented to have solid results. In particular, patients’ age was especially variable in pediatric trials, with one expanding enrolment beyond pediatric age (including patients with malignances up to 25 years old) [25]. Ventilatory strategy is also variable and, notably, often not protocolized and left to the decision of attending physicians in neonatal trials. Table 1 summarizes the characteristics of surfactant preparations trialed for ARDS in children and neonates as bolus administration or broncho-alveolar lavage. Six natural and one synthetic surfactant have been used. Animal-derived surfactants were either bovine or porcine and produced with various techniques. Surfactant preparations have extremely variable phospholipid profile and protein concentrations [26]. The synthetic surfactant (lucinactant) contains a synthetic peptide (KL_4_ or sinapultide) designed to mimic the sequence pattern of amphipathic helix of SP-B. Sinapultide is a 21-amino-acid hydrophobic synthetic peptide consisting of four leucine (L) and a lysine (K) repeating units. Moreover, it has a simple phospholipid profile, consisting of only dipalmitoylphosphatidylcholine, 1-palmitoyl-2-oleoyl-sn-glycero-3-phosphoglycerol and palmitic acid.

**Table 1:** Characteristics of surfactants trialed for ARDS, in children and neonates as bolus and/or broncho-alveolar lavage

Chemical name	Manufacturer	Country	Origin	Production method	PL (mg/mL)	Tested in children	Tested in neonates
Beractant	Abbvie	USA	Bovine	Minced lung	25	Yes	Yes
Bovactant	Boehringer Ingelheim Pharma	Germany	Bovine	Lung lavage	45	Yes	No
Calfactant	ONY/Pneuma Pharmaceuticals	USA	Bovine (calf)	Lung lavage	35	Yes	No
Kelisu (Calf Surfactant for injection)	CR Double Crane	China	Bovine (calf)	Lung lavage	30	No	Yes
Lucinactant	Discovery Lab	USA	Chemical	Synthetic	30	Yes	No
Poractant-α	Chiesi Farmaceutici	Italy	Porcine	Modified minced lung	80	Yes	Yes
Surface	CENSA	Cuba	Porcine	Lung lavage	25	Yes	No

Abbreviations: PL: phospholipids

Tables 2 and 3 summarize the review results: eight and ten trials investigated the use of surfactant for ARDS in children [25, 27–33] and neonates [34–43], respectively. Notably, one manuscript was not considered because it was a subgroup *post hoc* analysis of another trial [44].

**Table 2:**
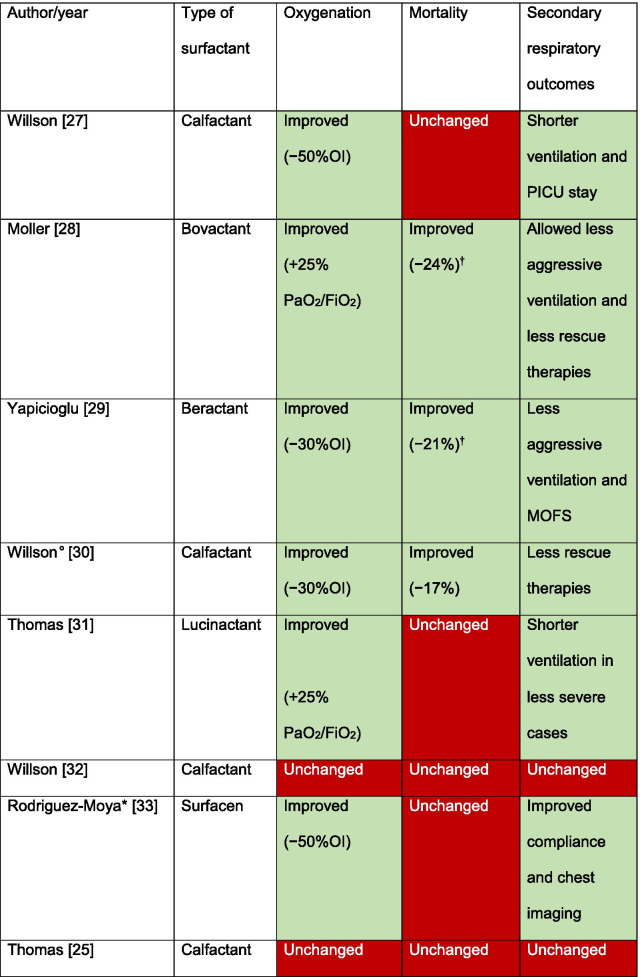
Synthesis of results: surfactant therapy in children

Boxes were colored in green or red if, for a given outcome, the surfactant treatment had a positive effect or not, respectively. Boxes were left blank where no data were available. No studies reported any biochemical or biophysical parameter

^†^Trend which does not reach significance threshold

°A further article is not considered, since it was a *post hoc* analysis of a subgroup enrolled in one of these trials [44]

^*^This trial investigated the administration of surfactant following recruitment maneuver *versus* standard care

Abbreviations: MOFS: multi-organ failure syndrome; PICU: pediatric intensive care unit

**Table 3:**
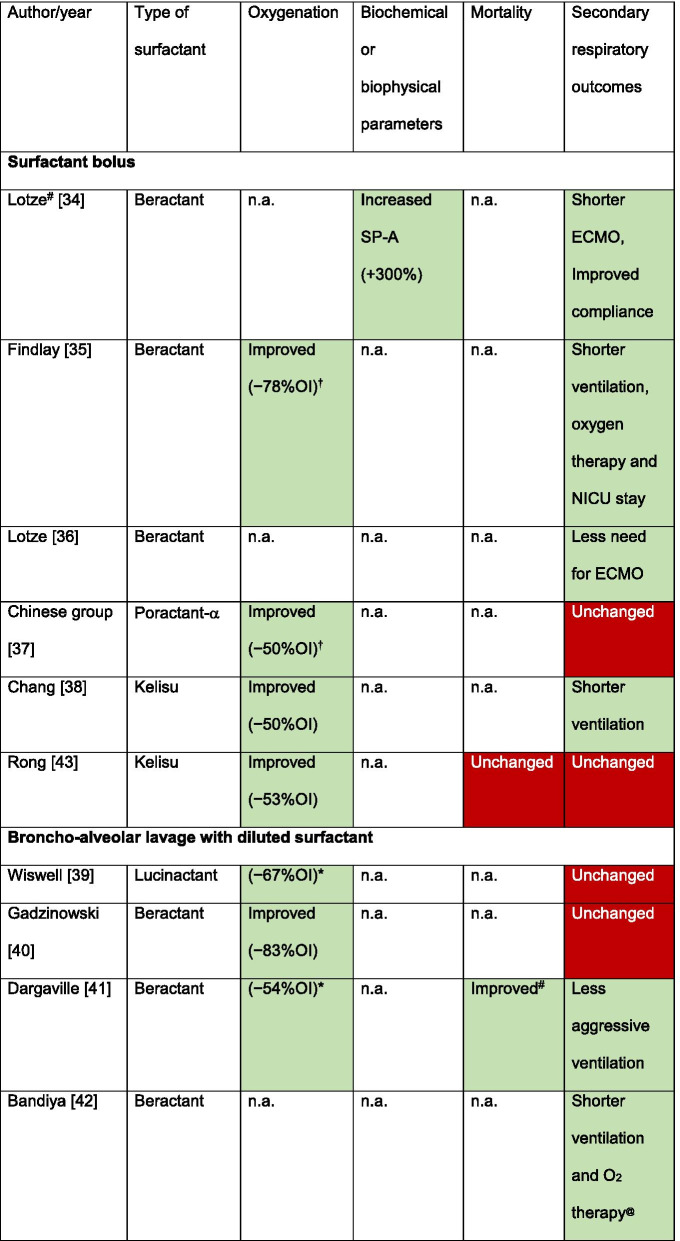
Synthesis of results: surfactant therapies in neonates

Boxes were colored in green or red if, for a given outcome, the surfactant treatment had a positive effect or none, respectively. Boxes were left blank where no data were available

^#^This study enrolled a mixed population of NARDS induced by meconium aspiration or sepsis but also non-ARDS conditions such as RDS or idiopathic persistent pulmonary hypertension of the neonate

^†^Same results with other oxygenation metrics; results obtained with up to 3 surfactant doses

^*^Trend which does not reach significance threshold. ^#^There was a significant reduction in the composite outcome mortality/need for extracorporeal life support. Raw mortality was not different between the two arms

^@^In addition to these outcomes, there was a trend for a clinically less severe respiratory distress

*ECMO* extra-corporeal membrane oxygenation, *PPHN* persistent pulmonary hypertension of the neonate, *RDS* neonatal respiratory distress syndrome (hyaline membrane disease)

None of the pediatric trials used the PALICC definition specific to PARDS [2]. Surfactant improved oxygenation in almost all these trials [25, 27–33], and mortality was lowered in 3 out of 8 trials [28–30]; other respiratory outcomes were also often ameliorated. Biochemical and biophysical parameters were not studied.

The Montreux definition of NARDS was released in 2017 and only one trial used it [43]. All the other neonatal trials enrolled patients with respiratory disorders appearing as NARDS but defined with variable criteria. Only one trial provided mortality data and showed improvement [41], and only one provided some biological or biophysical results [34]. Surfactant improved oxygenation in the 7 out of 10 trials [35, 37–40, 40–43]. Other outcomes were ameliorated in 6 trials [34–36, 38, 41, 42].

### Expert consensus

The following statements were approved:

1. There are sufficient preclinical and clinical data to support targeted research on surfactant therapies for PARDS and NARDS. Studies should be performed according to the currently available PARDS and NARDS definitions and considering the more recent knowledge on ARDS pathobiology *(strong agreement).*

Nowadays, the availability of appropriate clinical definitions for PARDS and NARDS [1, 2] (Additional file 1) represents an important step forward to improve ARDS care and recognizes that differences exist between patients of various ages and severity. Few trials have used these definitions, and their implementation is urgently warranted. Although both the PALICC and the Berlin [45] definitions could be used for adolescents or young adults [3], it is advisable to prefer the pediatric definition to allow further comparison and data aggregation [3]. Some neonatal respiratory disorders that may appear as NARDS have been variously defined before the release of the Montreux definition of NARDS [1]. Nonetheless, pathophysiology and biology are shared between these disorders and the use of an unique definition can speed up research and development of surfactant therapies for a wider number of patients [1]. Knowledge of ARDS and surfactant pathobiology has significantly advanced during recent years, and new concepts have been introduced, such as the role of secretory phospholipase A2 enzymes in the surfactant catabolism [11, 46], the vicious cycle connecting surfactant catabolism and inflammation [17], the deficiency and injury of surfactant proteins [23, 47], the variable cellular injury in direct (primary) and indirect (secondary) ARDS [14, 16] and the lower susceptibility of infant and juvenile compared to adult lung tissue [9]. These concepts should be taken into account in future trial design.

2. PARDS and NARDS should be considered as syndromes and should be preclinically studied according to key characteristics, such as direct (primary) or indirect (secondary) nature, clinical severity, infectious or non-infectious origin or patients’ age *(strong agreement).*

Several NARDS and PARDS experimental models are available to mimic these characteristics [48–51], but results of preclinical investigations cannot have the same value for all patients, since subtypes of PARDS and NARDS show relevant differences in pathobiology and pathophysiology. Several factors should be considered in pre-clinical models: trigger, animal size and the degree of lung development (i.e., patients’ age), presence of immunodeficiency or comorbidities, as well as the initial severity and the ventilatory strategy [1, 8, 52]. Unfortunately, the need of different preclinical model to mimic different clinical situations has often gone unnoticed and may have negatively influenced the perception of research results. Accumulating data show that these factors significantly influence the clinical course and outcomes: thus it is important to consider them before starting a clinical trial [14, 16, 17, 53–55]. Lung size and the volume available for aeration are also important as they will influence surfactant volume of distribution, the actual alveolar delivery and consequently the optimal dose and administration method [56]. The type of surfactant preparation and its concentration must also be considered since the administered volume may significantly influence the amount of phospholipids actually reaching the alveoli [56]. For these reasons, there is a clear need of translational studies focused on at least some of these factors [57]. In this context, studies aiming to clarify the optimal surfactant concentration and dose to be administered are urgently needed.

2. Explanatory should be preferred over pragmatic design for future clinical trials on PARDS and NARDS *(strong agreement)*.

This is the direct clinical counterpart of the previous point. ARDS is a very heterogeneous syndrome with various origins, presentations, severity, comorbidities and co-interventions. Trials enrolling broad groups of patients mixing all these factors are called pragmatic and seek a ‘real-world’ answer regarding an established intervention or refinement of current care [58]. This is not the case of surfactant and ARDS, since surfactant therapies are not yet standard of care for NARDS and PARDS and may represent a major improvement, rather than a minor refinement. Furthermore, although surfactant has a strong preclinical background suggesting its usefulness in ARDS, several concomitant clinical factors could influence response to treatment and outcomes [14, 16, 17, 53–55]. Patients’ age, type of ARDS, comorbidities, timing of the intervention, type and dose of surfactant, ventilatory policy and cointerventions are examples of the factors potentially important to stratify for. Trials with a highly specific selection or stratification are called explanatory and are more suitable and likely to provide significant results, although they are more complex and long-lasting [58]. There are at least 10 items to be considered in order to evaluate the pragmatism of a trial design [59]. Thus, future clinical trials should be as more explanatory as possible and focus on homogeneous groups of patients, according to the recently accumulated knowledge. In the UK, such trials are termed ‘efficacy and mechanism evaluation studies’ as they allow experimental mechanism evaluation that is unhindered by predefined trial outcome measures [60]. The choice of an explanatory design reflects the complexity of PARDS and NARDS, the need to consider them as multifaceted syndromes and to learn from correspondent preclinical models. When it comes to PARDS, patients’ age should also be restricted as much as possible, as it may significantly influence surfactant pharmacology and susceptibility to lung injury [9]. It is interesting to note that ventilation is the main co-intervention in these trials and was extremely variable and even non-protocolized in neonatal trials. This significantly reduces the trial quality and may have hindered the possibility to detect any effect of surfactant therapy.

A next step for a correct trial design would be a formal post hoc analysis of available data trying to identify which type of patients and ARDS should be the more likely to benefit from surfactant or which surfactant preparation should be preferred. According to a similar *post hoc* analysis of clinical trials conducted in adults [61] and to the recent knowledge on ARDS pathobiology and pharmacology [62], direct (primary) ARDS is more likely to benefit from surfactant treatments. In the future, we can imagine an even more individualized therapy by using genomic approaches or biomarkers of ARDS severity and type: the surfactant adsorption test [63–65] and the surfactant protein-D assay [66, 67] are two of the most advanced tools in this field. These, and other that may eventually come, can open the way for a precision medicine approach to ARDS surfactant therapy, as it is proposed in similar fields [68]. From a pharmacological point of view, preferably, an enhanced surfactant more resistant to phospholipase and inactivation has higher chances to be beneficial. A more concentrated surfactant seems also preferable, but the ideal dose and administration technique as well as the best strategy to spread it and increase alveolar delivery are still unknown and require specific studies.

4. Different clinical outcomes need to be chosen for PARDS and NARDS, according to the trial phase and design, type of trigger, severity class and/or surfactant treatment policy *(strong agreement).*

In general, trials must be conducted taking into consideration the most recent epidemiological data and a combination of clinically meaningful short- and long-term outcomes. For instance, it would be illogical to have mortality as a short-term outcome for mild PARDS or NARDS, as these are already subjected to relatively low mortality, while oxygenation, physiology parameters and burden of care measures are more suitable. As long-term outcomes, respiratory function measures may be preferred over pediatric quality of life measures which may be influenced by several other confounders [69].

Pathophysiological and biological plausibility is extremely important. Researchers should give priority to outcomes with a known direct pathophysiological link with the syndrome (such as short-term mortality, ventilator-free days and indices of gas exchange or oxygenation) [70–72]. Conversely, clinical endpoints that do not have a direct and clear connection with PARDS or NARDS should not be considered as primary outcomes. For instance, preterm neonates affected by NARDS may have negative long-term outcomes related to prematurity rather than to NARDS itself. Similarly, PARDS patients with malignancies may have a number of complications and negative outcomes because of their underlying disease rather than PARDS itself. Need for extracorporeal life support should only be considered if homogeneously available and defined in each recruiting center.

Finally, not only endpoints, but also the trajectory of disease should be considered in trial design. There are multiple benefits in doing so. For instance, oxygenation or ventilation metrics as well as lung mechanics parameters are repeated measures that lend themselves to multilevel (mixed effects) modeling. This will increase statistical power, which may be very important when studying particular populations in explanatory trials. This is also consistent with a personalized medicine approach as described above. Last but not least, multilevel modeling/trajectory analysis may adjust for the presence of other influencing factors such the availability of extra-corporeal life support or other co-interventions.

## Discussion

### Summary of the problem

It is clear that the current perspective of surfactant therapies for PARDS and NARDS was biased by significant trial flaws as well as lack of cross-disciplinary awareness and knowledge of ARDS pathobiology. Despite the importance of this subject, there has not been any consensus statement so far. As a consequence, trials have been initiated by different investigators, mainly without industry support or regulatory agencies contribution and without a coordination strategy. Meanwhile biological knowledge on surfactant and ARDS was increasing. Research in this field is difficult and will still present practical and logistical issues; however, the four recommendations represent a helpful tool. In fact, a consensus statement is important to put the knowledge in perspective, increase cross-disciplinary awareness and avoid errors from the past.

It is important to recall the history that led to these errors: surfactant replacement for RDS was preclinically and clinically tested for the first time in 1972 and 1980, respectively [73, 74]. At that time, RDS and ARDS were considered the two forms of respiratory distress exclusively typical of neonates and adults. As time has passed, it became apparent that ARDS was also occurring in children [75]. Meanwhile, due to improvements in perinatal care, the limit of viability has decreased so infants with RDS were more and more premature. Soon it has been realized that not all neonates with RDS responded to surfactant replacement with similar effectiveness [76] and so the existence of NARDS had been hypothesized [77]. Finally, PARDS and NARDS have been officially recognized with age-specific definitions modeled on the Berlin criteria [3], although earlier ARDS definitions were also applied on children [78]. The first attempts to treat PARDS with surfactant have shortly followed the enthusiasm of pediatricians using surfactant for RDS [79, 80]. Similarly, surfactant administration was tried for a number of neonatal ‘ARDS-like’ disorders (such as meconium aspiration syndrome, pneumonia, sepsis-related respiratory failure) [76]. However, these studies have been performed without: (1) clear definition of PARDS and NARDS, (2) key concepts of pathobiology and (3) clear drug development pathway.

### Clinical implications of biological research

Exogenous surfactant may be quickly inactivated and/or damaged by phospholipases: [81] commercially available surfactants do not contain crucial molecules providing anti-inflammatory or phospholipase-inhibitory activity, such as surfactant protein-A and -D or dioleoyl-phosphatidylglycerol (DOPG), and this may obviously reduce their efficacy [82, 83]. Club Cell secretory protein [84], human-recombinant surfactant protein-D [85], Nf-KB pathway inhibitors [86], DOPG [87] and varespladib [88, 89] are only some example of drugs that might fill this gap, protect surfactant and enhance its activity, while decreasing inflammation. Surfactants engineered to be more resistant to inactivation might also achieve the same objectives. While these molecules have not yet reached clinical use, it is not surprising that budesonide, a well-known synthetic steroid, had been administered using surfactant as carrier. This gave promising results in neonates with NARDS [90]. Also, budesonide decreases the expression of secretory phospholipase A2 [91] and has a clinically significant anti-inflammatory effect in preterm neonates at risk of broncho-pulmonary dysplasia [92]. Other surfactant-injuring agents have been well characterized in ARDS patients, and surfactant replacement might at least partially counterbalance the reduction of endogenous surfactant biophysical activity [23]. This could be measured at the bedside to personalize the dosing [47, 64]. In time, our knowledge about exogenous surfactant has increased and now we know that not all surfactant preparations are equal. Phospholipid profile and protein content are important for the clinical efficacy in neonates with RDS [26, 93], and it is likely that these characteristics would be relevant also in patients with PARDS and NARDS [23].

### Accuracy and difficulty of clinical trials design

Surfactant trials in PARDS and NARDS have not followed a clear drug development pathway, so far. Dose ranging trials are still lacking due to bias in funding allegedly ‘definitive studies.’ This is likely the main difficulty to design the adequate clinical trial. Others factors to be accurately considered are lung size and aeration, presence of phospholipases and other surfactant-injuring agents, timing of administration, ventilatory policy, resistance of surfactant to inactivation and presence of molecules enhancing its activity [88, 89, 94, 95]. Moreover, surfactant delivery is a complex physical phenomenon: any viscous fluid flowing over a surface leaves a coating film that increases in thickness as shear viscosity increases. Coating the conducting airways significantly reduces the amount of instilled surfactant really reaching the alveoli (i.e., the ‘coating cost’): the instilled volumes must be above the coating cost to deliver sufficient surfactant to the alveoli, and modeling studies show that surfactant doses trialed so far have overcome the coating cost only for neonates, but not for older patients [56]. This issue is extremely important, since too low doses may lack of efficacy, but too high doses may theoretically affect lung tissue inflammation and antibacterial defenses [96, 97].

Surfactant trials published so far have also quite different key features, and this made data aggregation difficult, especially for pediatric patients. Only recently, a multicenter study led to definition of sharing criteria to administer surfactant in PARDS as compassionate therapy [98]. Currently ongoing explanatory trials recruit homogeneous populations of infants with bronchiolitis-induced PARDS [60, 99]. These are examples of a correct step on the drug development pathway for a particular subgroup of patients.

The administration technique should also be deeply studied in order to ensure the best alveolar delivery. Bolus or broncho-alveolar lavage with diluted surfactant solutions may be used: the latter might be particularly efficacious in direct (primary) PARDS and NARDS [100, 101]. However, the technique is invasive and optimized surfactant bolus might provide equally significant benefits at least in some cases.

An adequate clinical research project should be based on recent pathobiology concepts and should start from finding the best dose and administration schedule/technique. Then, a trial enrolling direct (primary) PARDS or NARDS should be designed targeting meaningful clinical outcomes but also physiopathological and/or biological or biophysical measures. Enrolled patients should be as more homogeneous as possible, not only for the ARDS type but also for the other aforementioned factors.

## Conclusions

We advocate for well-designed preclinical and explanatory clinical studies to investigate the use of surfactant for PARDS and NARDS. Given the accumulating knowledge on ARDS biology, it is likely that surfactant therapies might be beneficial for PARDS and NARDS. Moreover, there is wide room for improving these therapies with the addition of drugs enhancing surfactant activity and/or reducing lung inflammation. It is also likely that previous inconsistent results would have been due to our lack of knowledge and misleading study designs. This field is an example of how preclinical knowledge can inform the clinical research pathway and how explanatory trials are needed to prevent losing promising therapies.

## Supplementary Information

**Additional file 1**. PARDS AND NARDS Definitions

**Additional file 2**. METHODS - Methods for literature review protocol and consensus methodology)

**Additional file 3**. ADDITIONAL RESULTS - Basic data of clinical trials

## Data Availability

All data generated or analyzed during this study are included in this published article and its supplementary information files.

## Abbreviations

ARDS: Acute respiratory distress syndrome
DOPG: Dioleoyl-phosphatidylglycerol
ECMO: Extra-Corporeal Membrane Oxygenation
MOFS: Multi-organ failure syndrome
NARDS: Neonatal acute respiratory distress syndrome
PALICC: Pediatric acute lung injury consensus conference
PARDS: Pediatric acute respiratory distress syndrome
PICU: Pediatric intensive care unit
PL: Phospholipids
RAND/UCLA: Research and Development/University of California, Los Angeles
RDS: Respiratory distress syndrome
