# Supplementary material for: Surfactant therapies for pediatric and neonatal ARDS: ESPNIC expert consensus opinion for future research steps
Source: Crit Care. 2021 Feb 22;25:75. doi: 10.1186/s13054-021-03489-6 (PMC7898495; doi:10.1186/s13054-021-03489-6)
Supplement: Supplementary file 1 — Additional file 1. PARDS AND NARDS Definitions [file 13054_2021_3489_MOESM1_ESM.docx]

**ADDITIONAL FILE N.1**

**PARDS AND NARDS DEFINITIONS**

**---**

**Surfactant therapies for pediatric and neonatal ARDS:**

**ESPNIC expert consensus opinion for future research steps**

Daniele De Luca (MD,PhD), Paola Cogo (MD,PhD), Martin C. Kneyber (MD,PhD),

Paolo Biban (MD), Calum Sample (MD), Jesus Perez-Gil (PhD), Giorgio Conti (MD),

Pierre Tissieres (MD,PhD) and Peter Rimensberger (MD,PhD)

**Montreux definition of NARDS [1]**

| **Montreux definition criteria** | |
| --- | --- |
| **Age** | Birth until 4 weeks, or 44 weeks post-menstrual age if born before 40 weeks’ gestation. |
| **Timing** | Acute onset (≤7 days) from a known or suspected clinical insult. |
| **Origin of edema** | Absence of congenital heart disease explaining pulmonary edema. |
| **Chest Imaging** | Diffuse, bilateral, irregular opacities or infiltrates or complete opacification of the lungs which are not fully explained by local effusions or atelectasis or congenital lung anomalies. Chest X-rays or lung ultrasound can be used if there is enough expertise.[2] |
| **Oxygenation Deficit** | Mild ARDS: OI≥4 and <8; Moderate ARDS: OI≥8 and <16; Severe ARDS: OI≥16. |
| **Exclusion Criteria** | |
| Respiratory distress syndrome (hyaline membrane disease) or transient tachypnoea of the neonate or congenital lung malformations | |
| Congenital heart disease explaining pulmonary edema | |
| Known genetic syndromes or chromosomopathies | |

**For the syndrome to be defined all criteria must be fulfilled**.

OI can be calculated by use of arterial or, if arterial values are unavailable, transcutaneous oxygen tension values, with appropriately calibrated transcutaneous devices. These devices should be used according to American Association of Respiratory Care guidelines;[3] a measurement at 44°C for max 10-15 minutes is usually harmless. If these devices are not available arterialized capillary blood gas analysis should be obtained, although the degree of heel warming cannot be controlled and is important for the reliability of PaO_2_ values measurements. In the case of persistent pulmonary hypertension of the neonate and patent ductus arteriosus, preductal PaO_2_ values should be used.

OI should be calculated with the most accurate measures available: thus, reducing airleaks if the patient is under non-invasive respiratory support and with the more accurate measurement of mean airway pressure.

The syndrome can be diagnosed at any gestational age or birthweight, provided that congenital lung anomalies, RDS, and TTN are excluded as primary respiratory disorder.

Criteria for the identification of RDS and TTN are as follows:

- RDS is defined as respiratory distress appearing within the first 24 h of life, with complete, sustained, and prompt response to surfactant or lung recruitment or both; additional non-mandatory criteria are lung imaging supporting the diagnosis or lamellar body counts ≤30 000/mm³, or both.
- TTN is defined as mild (Silverman score ≤3) respiratory distress appearing within the first 24 h of life and resolving within the first 72 h of life, needing treatment only with supplemental oxygen or nasal continuous positive airway pressure or both; additional non-mandatory criteria are lung imaging supporting the diagnosis or lamellar body counts >30 000/mm³, or both.

**Abbreviations**: ARDS=acute respiratory distress syndrome. RDS=respiratory distress syndrome. TTN=transient tachypnoea of the neonate. OI=oxygenation index.

**PALICC definition of PARDS [4]**


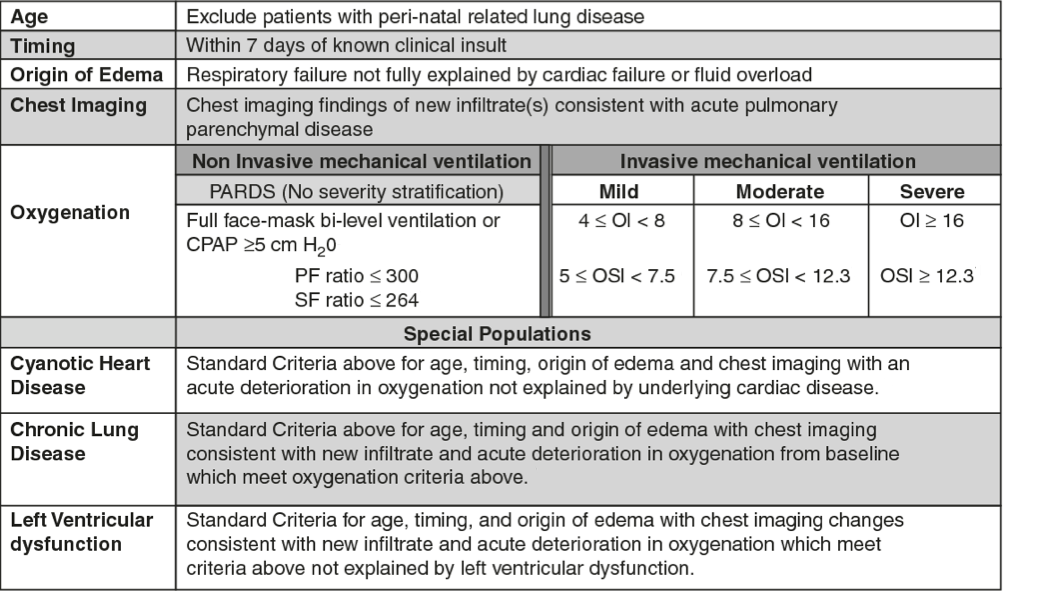


**For the syndrome to be defined all criteria must be fulfilled**.

**Abbreviations**: CPAP=continuous positive airway pressure. OI=oxygenation index. OSI=oxygen saturation index. PF ratio= PaO_2_/FiO_2_ ratio. SF ratio=SatO_2_/FiO_2_ ratio.

**REFERENCES**

1. De Luca D, van Kaam AH, Tingay DG, Courtney SE, Danhaive O, Carnielli VP, et al. The Montreux definition of neonatal ARDS: biological and clinical background behind the description of a new entity. Lancet Respir Med. 2017;5:657–66.

2. De Luca D, van Kaam AH, Tingay DG, Courtney SE, Danhaive O, Carnielli VP, et al. Lung ultrasound and neonatal ARDS: is Montreux closer to Berlin than to Kigali? – Authors’ reply. Lancet Respir Med. 2017;5:e32.

3. Restrepo RD, Hirst KR, Wittnebel L, Wettstein R. AARC Clinical Practice Guideline: Transcutaneous Monitoring of Carbon Dioxide and Oxygen: 2012. Respir Care. 2012;57:1955–62.

4. Khemani RG, Smith LS, Zimmerman JJ, Erickson S. Pediatric Acute Respiratory Distress Syndrome: Definition, Incidence, and Epidemiology. Pediatr Crit Care Med. 2015;16:S23–40.
