# Supplementary material for: Surfactant therapies for pediatric and neonatal ARDS: ESPNIC expert consensus opinion for future research steps
Source: Crit Care. 2021 Feb 22;25:75. doi: 10.1186/s13054-021-03489-6 (PMC7898495; doi:10.1186/s13054-021-03489-6)
Supplement: Supplementary file 2 — Additional file 2. METHODS - Methods for literature review protocol and consensus methodology) [file 13054_2021_3489_MOESM2_ESM.docx]

**ADDITIONAL FILE N.2**

**METHODS**

**---**

**Surfactant therapies for pediatric and neonatal ARDS:**

**ESPNIC expert consensus opinion for future research steps**

Daniele De Luca (MD,PhD), Paola Cogo (MD,PhD), Martin C. Kneyber (MD,PhD),

Paolo Biban (MD), Calum Sample (MD), Jesus Perez-Gil (PhD), Giorgio Conti (MD),

Pierre Tissieres (MD,PhD) and Peter Rimensberger (MD,PhD)

**LITERATURE SEARCH DETAILS AND CONSENSUS METHODOLOGY DETAILS**

***Literature search***

A protocol was agreed to determine the search modalities, eligibility criteria, data extraction and synthesis. Literature searches were conducted in PubMed for two categories: 1) ARDS in children (PARDS); and 2) in neonates (NARDS) according to the following standard key words or MeSH terms and limitations described in the main text. Search was conducted on PubMed using common key words and/or MeSH terms (see below, pag.5 in this additional file) and we also hand-searched references cited in the studies identified through the initial searches, and the authors’ personal archives. Abstracts, and where necessary full texts were reviewed by the whole group, duplicates and articles not meeting the eligibility criteria were removed. The studies were divided between those focusing on ARDS in children or neonates. No year or language restrictions were applied; we excluded “grey”, non-peer reviewed reports and preprints. Manuscripts not written in English were translated using Google translation service. Authors were contacted if some data were lacking or any clarification was needed.

The following clinical trial data were considered: study design, number of enrolled patients, basic population data, surfactant type, doses and administration method, oxygenation (measured with any metrics), mortality (defined as any of the following: intensive care unit or in-hospital mortality or composite outcomes mortality-extracorporeal life support) and any other respiratory outcome reported in the trials.

***Consensus methodology details***

The expert panel was composed as follows. Pediatric and neonatal intensivists expert in ARDS were selected amongst the European Society for Pediatric and Neonatal Intensive Care (ESPNIC) members. Experts were selected on the basis of their expertise in respiratory critical care and research track in the field during the last 10 years. Two independent non-ESPNIC member specialists, namely a biologist expert in surfactant biophysics (JPG), and a pediatric pulmonologist (MGS) were also added to increase multi-disciplinarity. Two co-authors (MCK and DDL) had relevant experience in guidelines development [1] and served as methodologists. The group size was decided by convenience sampling in order to facilitate the discussion. All invited experts agreed to participate.

One in-person meeting was organized and followed by several email discussions and videoconferences supported by ESPNIC. The panel used a Quaker-based consensus technique, which included open discussions with active listening and sharing of information and questions, until disputes (if any) were solved, with resulting ideas and solutions attributed to the whole group.[2] During the meetings the results of literature review and the panelists’ experience were discussed. Data were particularly discussed in light of ARDS pathophysiology and the biological plausibility for surfactant therapies. The whole panel identified the issues for future research steps and prepared them in form of bullet points. After 4 weeks, these were subjected to electronic, anonymous voting using a modified Research and Development/University of California, Los Angeles (RAND/UCLA) appropriateness scale, as previously done for ESPNIC mechanical ventilation guidelines.[1] The voting was organized by the ESPNIC secretariat using an online platform. Recommendations were scored from 1 (complete disagreement) to 9 (complete agreement) and the score was averaged after the elimination of one lowest and highest values. Recommendations were labelled as follows: “strong agreement” (if they had a mean score comprised between 7 and 9 and no score <7); “agreement” (mean score comprised between 7 and 9 and no score <4) or “disagreement” (mean score comprised between 1 and 3). Descriptive statistics were applied using Microsoft Excel 15.19.

**REFERENCES**

1. Kneyber MCJ, de Luca D, Calderini E, Jarreau P-H, Javouhey E, et al. on behalf of the section Respiratory Failure of the European Society for Paediatric and Neonatal Intensive Care. Recommendations for mechanical ventilation of critically ill children from the Paediatric Mechanical Ventilation Consensus Conference (PEMVECC).

Intensive Care Med. 2017;43:1764–80.

2. Quaker Foundations of Leadership. A comparison of Quaker-based consensus and Robert’s rules of order. Richmond (VI) USA: Earlham College; 1999.

For the literature search the following strings were used:

1. **for PARDS:**

Search with:

(("surface-active agents"[Pharmacological Action] OR "pulmonary surfactants"[Pharmacological Action] OR "surface-active agents"[MeSH Terms] OR ("surface-active"[All Fields] AND "agents"[All Fields]) OR "surface-active agents"[All Fields] OR "surfactant"[All Fields] OR "pulmonary surfactants"[MeSH Terms] OR ("pulmonary"[All Fields] AND "surfactants"[All Fields]) OR "pulmonary surfactants"[All Fields]) AND ARDS[All Fields]) AND ("child, preschool"[MeSH Terms] OR "child"[MeSH Terms:noexp] OR "adolescent"[MeSH Terms] OR "infant"[MeSH Terms:noexp])

and then with:

(("surface-active agents"[Pharmacological Action] OR "pulmonary surfactants"[Pharmacological Action] OR "surface-active agents"[MeSH Terms] OR ("surface-active"[All Fields] AND "agents"[All Fields]) OR "surface-active agents"[All Fields] OR "surfactant"[All Fields] OR "pulmonary surfactants"[MeSH Terms] OR ("pulmonary"[All Fields] AND "surfactants"[All Fields]) OR "pulmonary surfactants"[All Fields]) AND ("acute lung injury"[MeSH Terms] OR ("acute"[All Fields] AND "lung"[All Fields] AND "injury"[All Fields]) OR "acute lung injury"[All Fields])) AND ("child, preschool"[MeSH Terms] OR "child"[MeSH Terms:noexp] OR "adolescent"[MeSH Terms] OR "infant"[MeSH Terms:noexp])

and with:

("surface-active agents"[Pharmacological Action] OR "pulmonary surfactants"[Pharmacological Action] OR "surface-active agents"[MeSH Terms] OR ("surface-active"[All Fields] AND "agents"[All Fields]) OR "surface-active agents"[All Fields] OR "surfactant"[All Fields] OR "pulmonary surfactants"[MeSH Terms] OR ("pulmonary"[All Fields] AND "surfactants"[All Fields]) OR "pulmonary surfactants"[All Fields]) AND ARDS[All Fields] AND ("therapeutic irrigation"[MeSH Terms] OR ("therapeutic"[All Fields] AND "irrigation"[All Fields]) OR "therapeutic irrigation"[All Fields] OR "lavage"[All Fields])) AND ("child"[MeSH Terms:noexp] OR "infant"[MeSH Terms:noexp] OR "child, preschool"[MeSH Terms] OR "adolescent"[MeSH Terms])

and with:

(("surface-active agents"[Pharmacological Action] OR "pulmonary surfactants"[Pharmacological Action] OR "surface-active agents"[MeSH Terms] OR ("surface-active"[All Fields] AND "agents"[All Fields]) OR "surface-active agents"[All Fields] OR "surfactant"[All Fields] OR "pulmonary surfactants"[MeSH Terms] OR ("pulmonary"[All Fields] AND "surfactants"[All Fields]) OR "pulmonary surfactants"[All Fields]) AND ("acute lung injury"[MeSH Terms] OR ("acute"[All Fields] AND "lung"[All Fields] AND "injury"[All Fields]) OR "acute lung injury"[All Fields]) AND ("therapeutic irrigation"[MeSH Terms] OR ("therapeutic"[All Fields] AND "irrigation"[All Fields]) OR "therapeutic irrigation"[All Fields] OR "lavage"[All Fields])) AND ("child"[MeSH Terms:noexp] OR "infant"[MeSH Terms:noexp] OR "child, preschool"[MeSH Terms] OR "adolescent"[MeSH Terms])

1. **For NARDS:**

Search with:

("surface-active agents"[Pharmacological Action] OR "pulmonary surfactants"[Pharmacological Action] OR "surface-active agents"[MeSH Terms] OR ("surface-active"[All Fields] AND "agents"[All Fields]) OR "surface-active agents"[All Fields] OR "surfactant"[All Fields] OR "pulmonary surfactants"[MeSH Terms] OR ("pulmonary"[All Fields] AND "surfactants"[All Fields]) OR "pulmonary surfactants"[All Fields]) AND ("meconium aspiration syndrome"[MeSH Terms] OR ("meconium"[All Fields] AND "aspiration"[All Fields] AND "syndrome"[All Fields]) OR "meconium aspiration syndrome"[All Fields] OR ("meconium"[All Fields] AND "aspiration"[All Fields]) OR "meconium aspiration"[All Fields])

and then with:

("surface-active agents"[Pharmacological Action] OR "pulmonary surfactants"[Pharmacological Action] OR "surface-active agents"[MeSH Terms] OR ("surface-active"[All Fields] AND "agents"[All Fields]) OR "surface-active agents"[All Fields] OR "surfactant"[All Fields] OR "pulmonary surfactants"[MeSH Terms] OR ("pulmonary"[All Fields] AND "surfactants"[All Fields]) OR "pulmonary surfactants"[All Fields]) AND ("infant, newborn"[MeSH Terms] OR ("infant"[All Fields] AND "newborn"[All Fields]) OR "newborn infant"[All Fields] OR "neonatal"[All Fields]) AND ("acute lung injury"[MeSH Terms] OR ("acute"[All Fields] AND "lung"[All Fields] AND "injury"[All Fields]) OR "acute lung injury"[All Fields])

and with:

("surface-active agents"[Pharmacological Action] OR "pulmonary surfactants"[Pharmacological Action] OR "surface-active agents"[MeSH Terms] OR ("surface-active"[All Fields] AND "agents"[All Fields]) OR "surface-active agents"[All Fields] OR "surfactant"[All Fields] OR "pulmonary surfactants"[MeSH Terms] OR ("pulmonary"[All Fields] AND "surfactants"[All Fields]) OR "pulmonary surfactants"[All Fields]) AND ("infant, newborn"[MeSH Terms] OR ("infant"[All Fields] AND "newborn"[All Fields]) OR "newborn infant"[All Fields] OR "neonatal"[All Fields]) AND ARDS[All Fields]

and with

("surface-active agents"[Pharmacological Action] OR "pulmonary surfactants"[Pharmacological Action] OR "surface-active agents"[MeSH Terms] OR ("surface-active"[All Fields] AND "agents"[All Fields]) OR "surface-active agents"[All Fields] OR "surfactant"[All Fields] OR "pulmonary surfactants"[MeSH Terms] OR ("pulmonary"[All Fields] AND "surfactants"[All Fields]) OR "pulmonary surfactants"[All Fields]) AND ("therapeutic irrigation"[MeSH Terms] OR ("therapeutic"[All Fields] AND "irrigation"[All Fields]) OR "therapeutic irrigation"[All Fields] OR "lavage"[All Fields]) AND ("meconium"[MeSH Terms] OR "meconium"[All Fields])

and with:

("surface-active agents"[Pharmacological Action] OR "pulmonary surfactants"[Pharmacological Action] OR "surface-active agents"[MeSH Terms] OR ("surface-active"[All Fields] AND "agents"[All Fields]) OR "surface-active agents"[All Fields] OR "surfactant"[All Fields] OR "pulmonary surfactants"[MeSH Terms] OR ("pulmonary"[All Fields] AND "surfactants"[All Fields]) OR "pulmonary surfactants"[All Fields]) AND ("therapeutic irrigation"[MeSH Terms] OR ("therapeutic"[All Fields] AND "irrigation"[All Fields]) OR "therapeutic irrigation"[All Fields] OR "lavage"[All Fields]) AND ("infant, newborn"[MeSH Terms] OR ("infant"[All Fields] AND "newborn"[All Fields]) OR "newborn infant"[All Fields] OR "neonatal"[All Fields]) AND ("lung injury"[MeSH Terms] OR ("lung"[All Fields] AND "injury"[All Fields]) OR "lung injury"[All Fields])
