# Supplementary material for: Surfactant therapies for pediatric and neonatal ARDS: ESPNIC expert consensus opinion for future research steps
Source: Crit Care. 2021 Feb 22;25:75. doi: 10.1186/s13054-021-03489-6 (PMC7898495; doi:10.1186/s13054-021-03489-6)
Supplement: Supplementary file 3 — Additional file 3. ADDITIONAL RESULTS - Basic data of clinical trials [file 13054_2021_3489_MOESM3_ESM.docx]

**ADDITIONAL FILE N.3**

**ADDITIONAL RESULTS: basic data of clinical trials**

**---**

**Surfactant therapies for pediatric and neonatal ARDS:**

**ESPNIC expert consensus opinion for future research steps**

Daniele De Luca (MD,PhD), Paola Cogo (MD,PhD), Martin C. Kneyber (MD,PhD),

Paolo Biban (MD), Calum Sample (MD), Jesus Perez-Gil (PhD), Giorgio Conti (MD),

Pierre Tissieres (MD,PhD) and Peter Rimensberger (MD,PhD)

**Table 1. Basic data of trials of surfactant therapies in children with PARDS.**

Patients’ age has been expressed in months as mean (standard deviation) or median [25^th^-75^th^ percentile], calculated (if needed) as mean of the two study arms and rounded to the closest decimal. Percentage of direct ARDS has been rounded to the closest number. Administered volume is calculated as total volume injected, according to the administration schedule and to the averaged patients’ weight or, when this was unavailable, to the 50^th^ percentile of male children growth curve for the mean reported population age and rounded to the closest decimal. **Abbreviations**: FiO_2_: inspired oxygen fraction; n.a.: not available; PEEP: positive end-expiratory pressure; PIP: peak inspired pressure; Tv: tidal volume

* This study reported patients’ age for classes and this was spanned between 0 and 16 years.

| **Authors, year**  **[reference]** | **Age**  **(months)** | **Patients n.** | **Direct PARDS** | **Administered dose** | **Administered volume (mL)** | **Mode of administration** | **Ventilation strategy** |
| --- | --- | --- | --- | --- | --- | --- | --- |
| *Willson, 1999*  *[27]* | 57 (65) | 42 | 93% | 2.8 g/m^2^ (80 mL/m^2^) body surface up to three of doses of | n.a. | Bolus with patient rotation.  No recruitment manoeuvres. Ventilatory parameters (beyond FiO_2_) unchanged during administration | Tv 10-12 mL/Kg or PIP <30 cmH_2_O, PEEP <5 cmH2O, FiO_2_<0.6 and change frequency to titrate ventilation – Targets: PaCO_2_<60 mmHg,  pH ≥7.25 |
| *Moller, 2003*  *[28]* | 46 [0-156] | 35 | 74% | 100 mg/Kg (2.2 mL/kg) up to two doses | 41-82 | Bolus with patient rotation.  No recruitment manoeuvres.  Ventilatory parameters unchanged during administration. | Tv<10 mL/Kg or PIP <30 cmH_2_O, PEEP <5 cmH2O – Targets: PaCO_2_<65 mmHg, pH >7.2 |
| *Yapicioglu, 2003*  *[29]* | 72.5 (56) | 36 | 25% | 150 mg/kg (6 mL/kg) up to two doses | 102-204 | Bolus with patient rotation. No other details | No details on ventilatory policy - Targets: SatO_2_: 90-95%, PaO_2_>60 mmHg, pH: 7.30-7.35 |
| *Willson, 2005*  *[30]* | 83 (77) | 152 | 58% | Two doses of 2.8 g/m^2^ (80 mL/m^2^) body surface or 105 mg/kg for infants <10 kg | n.a. | Bolus with patient rotation and neuromuscular blockade. No recruitment manoeuvres. Ventilatory parameters (beyond FiO_2_) unchanged during administration | Tv<8 mL/Kg and PIP <40 cmH_2_O, FiO_2_<0.6 – Targets: PaCO_2_<60 mmHg |
| *Thomas, 2012*  *[31]* | 5.8 (5.6) | 165 | 95% | 175 mg/kg (5.8 mL/kg) up to two doses | 36-72 | Bolus with patient rotation.  No recruitment manoeuvres. Ventilatory parameters adjusted during administration according to patients’ conditions. | Tv=6 mL/Kg and use PEEP/FiO_2_ table and change frequency to titrate ventilation – Targets: SatO_2_: >88%, PaO_2_: 55-80 mmHg, pH: 7.25-7.45 |
| *Willson, 2013*  *[32]* | 74 (71) | 109 | 100% | 30 mg (≈1 mL) per cm of height (in two equal aliquots) or 100 mg/kg (3.3 mL/kg) for infants <10 kg | n.a. | Bolus with patient rotation.  No recruitment manoeuvres. Ventilatory parameters (beyond FiO_2_) unchanged during administration | Use PEEP/FiO_2_ table and change frequency to titrate ventilation – Targets: SatO_2_: 85-92%, PaO_2_: 50-80 mmHg, pH: 7.25-7.45 |
| *Rodriguez-Moya*  *2017 [33]* | n.a.* | 31 | 71% | Nine dose of 100 mg/kg (4 mL/kg) each | n.a. * | Bolus without patient rotation. No other details | Use “best PEEP” (not specified in detail) – Targets: SatO_2_: 88-90%, PaO_2_: >60 mmHg, PaCO_2_<80 mmHg, pH: >7.20 |
| *Thomas, 2018*  *[25]* | 149 (68) | 43 | 53% | Two doses of 2.8 g/m^2^ (80 mL/m^2^) body surface or 105 mg/kg (3 mL/kg) for infants <10 kg | 107-215 | Bolus without patient rotation. No other details | Tv ≤6 mL/Kg or plateau pressure ≤30 cmH_2_O, FiO_2_≤0.6 – Targets: SatO_2_: >88%, PaO_2_: >60 mmHg, PaCO_2_<80 mmHg, pH: >7.25 |

**Table 2. Basic data of trials of surfactant therapies in newborn infants with NARDS.** Gestational age has been expressed in weeks as mean (standard deviation) or median [25^th^-75^th^ percentile], calculated (if needed) as mean of the two study arms and rounded to the closest decimal. Percentage of direct NARDS has been rounded to the closest number. Administered volume is calculated as total volume injected, according to the administration schedule and using the averaged patients’ weight and rounding to the closest decimal. Some data on ventilatory strategy were neither reported on the original article, nor they were available by contacting authors. **Abbreviations**: AC: assisted control ventilation; HFOV: high frequency oscillatory ventilation; FiO_2_: inspired oxygen fraction; MAS: meconium aspiration syndrome; n.a.: not available; PEEP: positive end-expiratory pressure; PIP: peak inspired pressure; SIMV: synchronized intermittent mandatory ventilation.

* This study reported gestational age for classes and this was spanned 34 and 40 weeks.

^#^ This study did not report gestational age but enrolled patients born at ≥ 35 weeks’ gestation

| **Authors, year**  **[reference]** | **Age**  **(weeks)** | **Patients**  **n.** | **Direct NARDS** | **Administered dose** | **Administered volume (mL)** | **Mode of administration** | **Ventilation strategy** |  |
| --- | --- | --- | --- | --- | --- | --- | --- | --- |
| ***Surfactant bolus*** | | | | | | | | |
| *Lotze, 1993*  *[34]* | 39.5 (2) | 56 | 61% | 100 mg/kg (4 mL/kg) up to 4 doses | 14-56 | Bolus without patient rotation | n.a. |  |
| *Findlay, 1996*  *[35]* | 39.9 (0.4) | 40 | 100% (MAS) | 150 mg/kg (6 mL/kg) up to 4 doses | 20-82 | Bolus without patient rotation using a side port access side of the endotracheal tube | n.a. |  |
| *Lotze, 1998*  *[36]* | 39 (1.8) | 328 | 51% | 100 mg/kg (4 mL/kg) up to 4 doses | 14-56 | Bolus without patient rotation | At the discretion of attending physicians |  |
| *Chinese group*  *2005*  *[37]* | 39.8 (1.6) | 61 | 100% (MAS) | 200 mg/kg (2.5 mL/kg) followed by other three doses of 200 and 100 mg/kg (1.25 mL/kg) up to 4 doses | 8.5-30 | Bolus without patient rotation with brief ventilation on 100% oxygen. | n.a. |  |
| *Chang, 2016*  *[38]* | n.a.* | 136 | n.a. | 70-100 mg/kg  (2.3-3.3 mL/kg) | 6-8.7 | n.a. | SIMV or AC as follows: PIP: 18-25 cmH_2_O, PEEP: 4-6 cmH_2_O, frequency 35-60, FiO_2_: 0.3-0.6  HFOV as follows: Paw: 10-14 cmH_2_O, amplitude: 35-45 cmH_2_O, frequency 9-11 Hz, FiO_2_: 0.3-0.6 -  Targets: good blood gas analysis |  |
| *Rong, 2020*  *[43]* | 37.6 (2.3) | 328 | 100% (pneumonia) | 100 mg/kg (3.3 mL/kg) | 29.7 | Bolus without patient rotation | n.a. |  |
| ***Broncho-alveolar lavage with diluted surfactant*** | | | | | | | | |
| *Wiswell, 2002*  *[39]* | 39.6 (1.5) | 22 | 100% (MAS) | Sequential lavages with 2.5 OR 10 mg/mL solutions | Multiple aliquots of 8 mL/kg | Non-bronchoscopic broncho-alveolar lavage per each lung, with patient rotation, during expiratory pause with unchanged PEEP | At the discretion of attending physicians |  |
| *Gadzinowski, 2008*  *[40]* | n.a.^#^ | 13 | 100% (MAS) | Lavage with 5 mL/ml solution | 15 mL/kg (in 4 aliquots) | Non-bronchoscopic broncho-alveolar lavage per each lung, with patient rotation, with unchanged ventilator parameters | Not available |  |
| *Dargaville, 2011*  *[41]* | 39.5 [38-41] | 66 | 100% (MAS) | Two lavages with 5 mL/ml solution | 30 mL/kg (in 2 aliquots) | Non-bronchoscopic broncho-alveolar lavage per each lung, with patient rotation, with unchanged ventilator parameters | At the discretion of attending physicians |  |
| *Bandiya, 2019*  *[42]* | 38 [38-39] | 60 | 100% (MAS) | Lavage with 5 mL/ml solution | 20 mL/kg (in 4 aliquots) | Non-bronchoscopic broncho-alveolar lavage per each lung, with patient rotation, with unchanged ventilator parameters | At the discretion of attending physicians |  |
